# Chemoradiotherapy by intensity-modulated radiation therapy with simultaneous integrated boost in locally advanced or oligometastatic non-small-cell lung cancer—a two center experience

**DOI:** 10.1007/s00066-021-01756-7

**Published:** 2021-03-16

**Authors:** Frederick Mantel, Elena Müller, Philip Kleine, Marcus Zimmermann, Florian Exner, Anne Richter, Stefan Weick, Serge Ströhle, Bülent Polat, Stefan Höcht, Michael Flentje

**Affiliations:** 1 Department of Radiation Oncology, University of Würzburg, Josef-Schneider-Str. 11, 97080 Würzburg, Germany; 2 Xcare Practice for Radiation Therapy, Saarlouis, Germany

**Keywords:** Image-guided radiation therapy, Thoracic cancer, Hypofractionation, Multimodal therapy, Local control

## Abstract

**Purpose:**

Integrating moderate hypofractionation to the macroscopic tumor with elective nodal irradiation while sparing the organs at risk (OAR) in chemoradiotherapy of locally advanced non-small-cell lung cancer.

**Methods:**

From 2010–2018, treatment, patient and tumor characteristics of 138 patients from two radiation therapy centers were assessed. Chemoradiotherapy by intensity-modulated radiation therapy (IMRT) with a simultaneous integrated boost (SIB) to the primary tumor and macroscopic lymph node metastases was used.

**Results:**

A total of 124 (90%) patients received concurrent chemotherapy. 106 (76%) patients had UICC (Union for International Cancer Control) stage ≥IIIB and 21 (15%) patients had an oligometastatic disease (UICC stage IV). Median SIB and elective total dose was 61.6 and 50.4 Gy in 28 fractions, respectively. Furthermore, 64 patients (46%) had an additional sequential boost to the primary tumor after the SIB-IMRT main series: median 6.6 Gy in median 3 fractions. The median cumulative mean lung dose was 15.6 Gy (range 6.2–29.5 Gy). Median follow-up and radiological follow-up for all patients was 18.0 months (range 0.6–86.9) and 16.0 months (range 0.2–86.9), respectively. Actuarial local control rates at 1, 2 and 3 years were 80.4, 68.4 and 57.8%. Median overall survival and progression-free survival was 30.0 months (95% confidence interval [CI] 23.5–36.4) and 12.1 months (95% CI 8.2–16.0), respectively. Treatment-related toxicity was moderate. Radiation-induced pneumonitis grade 2 and grade 3 occurred in 13 (9.8%) and 3 (2.3%) patients.

**Conclusions:**

Chemoradiotherapy using SIB-IMRT showed promising local tumor control rates and acceptable toxicity in patients with locally advanced and in part oligometastatic lung cancer. The SIB concept, resulting in a relatively low mean lung dose, was associated with low numbers of clinically relevant pneumonitis. The overall survival appears promising in the presence of a majority of patients with UICC stage ≥IIIB disease.

## Introduction

The therapeutic intent in locally advanced non-small-cell lung cancer (NSCLC) is curative, and radiation therapy (RT) is a cornerstone of treatment. RT is most commonly combined with simultaneous platinum-based chemotherapy, as a concurrent approach has been shown to be superior to a sequential approach with regard to local control (LC) and overall survival (OS) [1]. However, the curative intention of chemoradiotherapy (CRT) remains challenging. Progression-free survival (PFS) after 1 and 2 years has been reported to be approximately 40–50% and 20–30%, respectively. The addition of consolidation chemotherapy does not prolong PFS or OS [2]. By contrast, consolidation immunotherapy by the anti-programmed death ligand 1 antibody durvalumab resulted in significantly longer progression-free and overall survival than placebo and represents the current standard of care for patients without progressive disease after CRT [3, 4]. While most patients suffer from distant progression after CRT, 30–40% of patients experience local failure after 2 years [5]. Intensity-modulated radiation therapy (IMRT) combined with image-guided radiation therapy (IGRT) enables the use of moderate hypofractionation to the gross tumor volume (GTV) combined with elective nodal irradiation and possibly reduces exposure of uninvolved lung areas. Here, we report on a two-center experience of chemoradiotherapy for locally advanced NSCLC using simultaneous-integrated boost IMRT (SIB-IMRT).

## Methods

### Study patients

We performed a retrospective analysis of 138 patients treated from 2010–2018 for locally advanced and in part oligometastatic NSCLC at the University of Würzburg (institution 1) and the Xcare practice for Radiation Therapy, Saarlouis (institution 2) using a nearly identical treatment strategy. All patients gave informed consent and we obtained approval from the ethics committee for this study. In all, 133 patients (96.4%) underwent positron emission tomography (PET) staging. By default, clinically suspect mediastinal or supraclavicular lymph nodes were biopsy-confirmed by endobronchial ultrasound (EBUS) or mediastinoscopy, unless obvious clinical signs of lymph node metastases (e.g. mediastinal lymph node bulk) were present. All patients with oligometastatic disease were treated in institution 1. Oligometastatic disease included patients with synchronous pleural nodules, contralateral pulmonary lesions or a maximum of two synchronous extrathoracic metastases including brain, liver, bone, adrenal gland and soft tissue. Oligometastatic lesions were treated by resection and/or dose intensified radiation therapy.

As the 8th edition of the UICC (Union for International Cancer Control) TNM classification was released in the beginning of 2017, we performed a retrospective verification of the UICC TNM tumor classification and a reclassification according to the 7th and 8th edition for all patients, respectively.

### Treatment planning and radiotherapy

Patients were treated with concurrent chemoradiotherapy using a SIB-IMRT technique with image-guidance by linac-integrated cone-beam computed tomography (CBCT). In institution 1 daily CBCT was acquired for the first five fractions for generation of a mean value for set-up error and then every third fraction in case detected set-up errors did not exceed 3 mm; otherwise a CBCT was acquired prior to the subsequent fraction. In case a correction of rotational errors was reasonable, daily CBCT was performed. The decision for correction of rotational errors was made at the discretion of the treating radiation oncologist. Institution 2 used IGRT with daily CBCT for all cases. Treatment planning computed tomography (CT) with a slice-thickness of 3 mm was acquired in mid-ventilation and supine position with elevated arms in a vacuum mattress or another individually adjustable immobilization device. An additional 4D-planning-CT was performed for all patients and the end-exhalation and end-inhalation phase were reconstructed and used as secondary image files in the planning software for consideration of breathing-related tumor motion in institution 1, whereas in institution 2 4D-planning-CTs were predominantly used for tumors located in the lower lobes with expected higher mobility. Pinnacle (Philips Radiation Oncology Systems, Milpitas, CA, USA) was used for treatment planning for an Elekta Synergy/Versa HD platform equipped with Agility Head/MLC (Elekta Oncology Systems, Crawley, UK). The gross tumor volume (GTV) included the macroscopic primary tumor and macroscopic lymph node metastases. Lymph nodes were considered involved when enlarged (short axis in CT > 1 cm), showed an elevated standardized uptake value (SUV) (≥2.5) or when tumor infiltration was proven by EBUS cytology. However, we followed a “generous” approach and usually included also lymph nodes, e.g., with borderline elevated SUV or those in close vicinity to proven metastatic nodes. F-18 fluorodeoxyglucose positron emission tomography (FDG-PET) information was used for GTV delineation in 133 patients by coregistration of the PET with the planning CT. FDG-PET was acquired in the same position as the planning CT, i.e., in supine position with elevated arms. The GTV was contoured in free breathing. In case of 4D-CT acquisition the GTV was delineated additionally in the end-exhalation and end-inhalation position to generate an internal target volume (ITV) as the sum of the GTVs. No additional margin was added to the GTV or ITV to generate the clinical target volume (CTV) SIB. The elective CTV of hilus and mediastinum included the lymph node stations neighboring metastatic involved node regions. In institution 1 regions with a risk of metastatic involvement greater than 10% according to Giraud et al. [6] and the supraclavicular stations, in case of upper lobe primary tumor or mediastinal lymph node metastases in stations 1 or 2, were included. Contouring was done according to the atlas published by Chapet et al. [7]. An additional margin of 3–5 mm was added to the CTV SIB to generate the planning target volume (PTV) SIB. The elective target volume (PTV) included the CTV SIB and the elective CTV with an addition of 5–10 mm. Patients received a follow-up planning-CT on a regular basis after 30 Gy for consideration of tumor response or re-ventilation in case of atelectatic lung regions in institution 1, or earlier, if CBCT showed significant changes in comparison to the initial planning CT, while daily CBCTs were used for deciding on the necessity to do a re-planning in institution 2. If tumor shrinkage was present, which in the discretion of the treating radiation oncologist enabled for a reduction of the radiation field, target volume and plan-adaption were performed accordingly to achieve better organ-at-risk (OAR) sparing. Both PTV and boost treatment dose was prescribed to the encompassing 95% isodose with a tolerance range of ±2%, typically with a single dose of 1.8 Gy to the PTV and 2.2 Gy to the PTV SIB in 28 fractions. Lungs, the heart, esophagus and spinal canal were contoured as OAR according to the NRG/RTOG contouring atlas [8]. Lung dose constraints were 30 Gy D_mean_, a V_5_ _Gy_ of 90% and 10 Gy D_mean_, a V_5_ _Gy_ of 50% for the ipsilateral and contralateral lung, respectively. D_mean_ for both lungs together should not exceed 17 Gy, but higher doses could be accepted at the discretion of the treating radiation oncologist. Heart D_mean_ was limited to 10 Gy and D_01_ to 35 Gy but target volume coverage had priority. Dose to the esophagus was limited to a D_10_ of 60 Gy. For the spinal canal, D_1_ _cm3_ was constrained to 45 Gy. IGRT was used in every patient based on the regular acquisition of CBCT with online correction for set-up errors.

### Chemotherapy

Concurrent chemotherapy consisted of cisplatin 20 mg/m^2^ body surface area (BSA), days 1–4, 29–32 and vinorelbine 15 mg/m^2^ BSA, days 1, 8, 15, 29, 36, 43. Alternatively, chemotherapy with weekly carboplatin area under the curve (AUC) 2 and paclitaxel 50 mg/m^2^ BSA was used. Paclitaxel mono weekly 60 mg/m^2^ BSA or carboplatin mono weekly AUC2 was applied in frail patients. Patients were selected for concomitant CRT on the basis of Karnofsky performance status (KPS), age, and comorbidities at the discretion of the treating radiation oncologist. Patients not eligible for chemotherapy were treated with SIB-IMRT only.

### Follow-up and endpoint assessment

Time intervals were calculated from the date of RT completion. During follow-up (FU) CT scans were performed at each FU visit every 3 months in the first 2 years and every 6 months thereafter. Locoregional failure was defined using response evaluation criteria in solid tumors (RECIST) [9]. Local control was defined as freedom from tumor progression within the RT field. Regional control was defined as freedom from tumor progression at all mediastinal sites outside the RT field and all pulmonary sites adjacent to the primary tumor but outside the RT field. In some cases PET was performed and local failure was defined in consultation with the specialist of nuclear medicine. Radiological FU was missing for 9 patients due to early death during or after treatment and for 1 patient who was lost to FU before the first planned radiological assessment. Acute and late radiation-induced toxicity was scored by using common terminology criteria for adverse events (CTCAE) version 4.0. Acute toxicities were assessed weekly during RT and 6 weeks afterwards. Late toxicities were recorded every 3 months in the first year and every 6 months thereafter.

### Statistical analysis

SPSS version 25 (SPSS, Inc.) was used for statistical analyses. Kaplan–Meier analyses were performed for local tumor control (LC), progression-free survival (PFS) and overall survival (OS) for the entire patient cohort and separately for the subgroup of patients with UICC stage III treated with concurrent CRT. Comparisons of LC in patients receiving CRT versus RT alone and of OS and PFS in stage IV versus earlier stage patients were carried out by log-rank tests. Univariate analysis for the likelihood of death or local recurrence was performed using a logistic regression. Cutoff points were chosen for UICC stage, total dose, GTV, PTV SIB, PTV, D_mean_ lungs based on receiver operating characteristic analyses. All variables that were statistically significant or trended to significance (*p* ≤ 0.1) were included in the multivariate analysis. For multivariate analysis a logistic regression with the stepwise forward method was used. A *p* value ≤ 0.05 was considered statistically significant.

## Results

From 2010–2018, 138 patients suffering from locally advanced and in part oligometastatic NSCLC were treated in the department of radiation oncology, University of Würzburg and the Xcare practice for Radiation Therapy, Saarlouis with an identical definitive SIB-IMRT protocol. Patient and treatment characteristics are listed in Table 1. Median age was 63 years and 68% were male. Most common histology was adenocarcinoma (58%) and squamous cell carcinoma (38%). According to the UICC TNM classification in its 7th edition, 91 (66%) patients had UICC stage ≥IIIB, among them 21 (15%) patients with oligometastatic disease (UICC stage IV). After reclassification of all 138 patients according to the 8th edition of the UICC TNM classification, 106 (76%) patients had UICC stage ≥IIIB and 60 (43%) had stage ≥IIIC. In all, 64 (46%) patients had nodal stage 3 (N3). Fourteen (10%) patients were classified as stage IVA and 7 (5%) patients as stage IVB, respectively. A total of 133 (96.4%) patients underwent diagnostic PET staging. Furthermore, 124 (90%) patients received concurrent chemotherapy predominantly consisting of a platinum-based doublet. Fourteen (10%) patients were physically unfit for chemotherapy treatment and were treated with SIB-IMRT only. Median SIB-IMRT boost and elective PTV total dose was 61.6 and 50.4 Gy, respectively, in median 28 fractions. Sixty-nine (50%) patients also received a plan adaption during the SIB-IMRT course due to tumor response or changes in tumor location (e.g., reventilation of atelectasis or pleural effusion). On average 27% of the PTV was included in the SIB-escalated dose region. The dose planning constraints were reliably met over a large range of PTV volumes (55–2400 ml, median 689 ml). After completion of the SIB-IMRT course an additional sequential boost to the primary tumor was applied in 64 patients (46%) on basis of a new planning CT in order to achieve further tumor regression. The sequential boost consisted of median 6.6 Gy in 3 fractions. This resulted in a median total dose to the primary tumor of 63.8 Gy (range 54.0–71.6 Gy) for all patients. Median cumulative mean lung dose was 15.6 Gy (range 6.2–29.5 Gy). Concurrent chemotherapy was administered per protocol in 104 patients (85%), a dose reduction became necessary in 6 patients (5%) and chemotherapy had to be stopped in 12 patients (10%).

**Table 1:** Patient and treatment characteristics

Parameter		
*Age, median (range), years*	63	(37–89)
*Sex, No. (%)*		
Female	44	(32)
Male	94	(68)
*Karnofsky performance status, median (range), %*	90	(50–100)
*Body mass index, median (range), kg/m*^*2*^	25	(16–46)
*FEV*_*1*_* median (range), liter*	2.1	(0.6–4.2)
*FEV*_*1*_* % of target, median (range)*	74	(18–127)
*Histology, No. (%)*		
Adenocarcinoma	80	(58)
Squamous cell carcinoma	53	(38)
Large-cell neuroendocrine carcinoma	4	(3)
Adenocystic carcinoma	1	(1)
*Grading, No. (%)*		
G1	1	(1)
G2	33	(33)
G3	64	(64)
G4	2	(2)
*UICC tumor stage (7th edition), No. (%)*		
2A	1	(1)
2B	2	(1)
3A	44	(32)
3B	70	(51)
4	21	(15)
*UICC tumor stage (8th edition), No. (%)*		
2A	1	(1)
2B	2	(2)
3A	29	(21)
3B	46	(33)
3C	39	(28)
4A	14	(10)
4B	7	(5)
*Cumulative total dose, median (range), Gy*	63.8	(54–71.6)
*Total No. of fractions, median (range)*	29	(25–33)
*Total dose of SIB-IMRT, median (range), Gy*		
Elective target volume (PTV)	50.4	(41.4–57.1)
Simultaneous integrated boost (SIB)	61.6	(50.6–69.0)
No. of fractions	28	(20–32)
*Sequential boost, No. (%)*	64	(46)
Total dose, median (range), Gy	6.6	(2–20)
No. of fractions	3	(1–5)
*GTV, median (range), cm*^*3*^	95.9	(14.7–891.7)
*SIB, median (range), cm*^*3*^	186.6	(24.9–1361.3)
*PTV, median (range), cm*^*3*^	689.1	(55.4–2401.9)

*FEV*_*1*_ Forced expiratory volume in 1 second, *UICC* Union for International Cancer Control, *Gy* Gray, *No.* Number, *SIB-IMRT* Simultaneous integrated boost–intensity-modulated radiation therapy, *PTV* Planning target volume, *GTV* Gross tumor volume

**Fig. 1:**
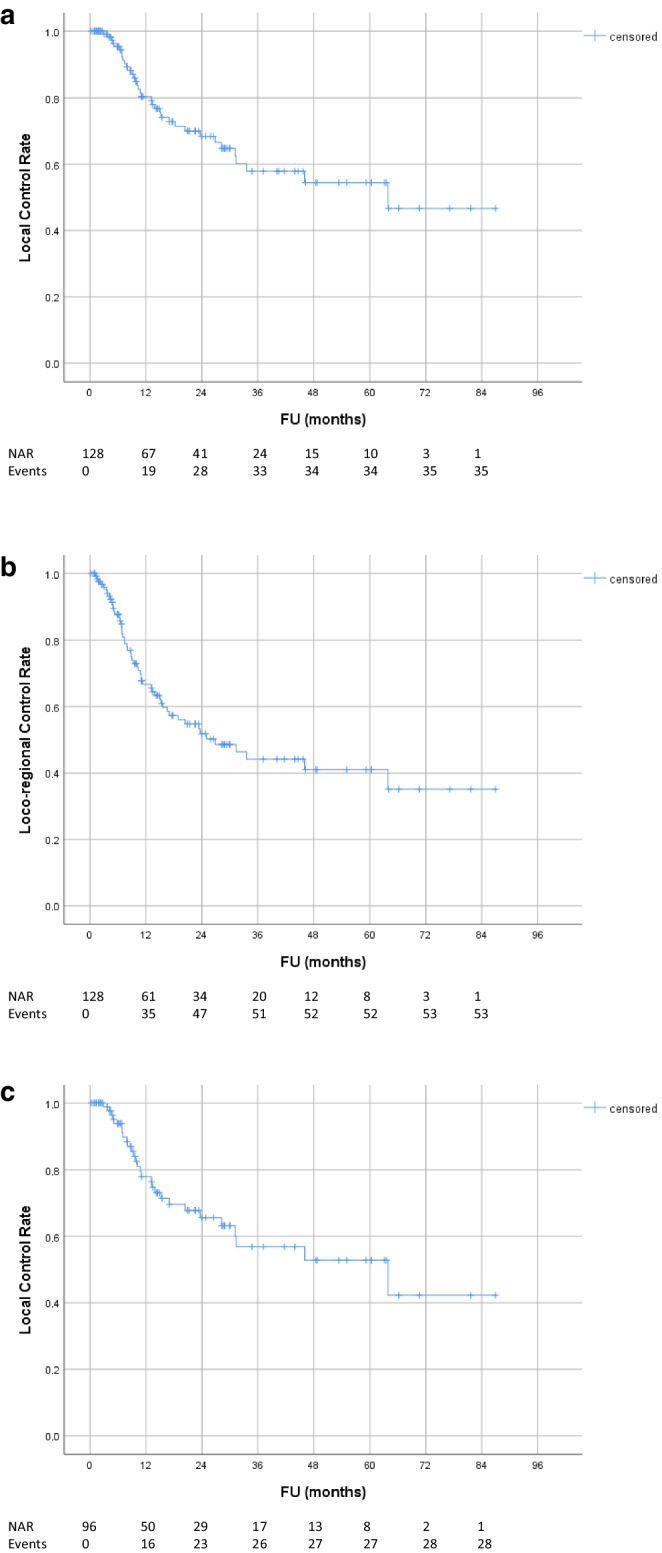
Tumor control rate: **a** local (in-field) tumor control; **b** locoregional control (in- and out-field); **c** local (in-field) tumor control for subgroup of patients with UICC stage III treated with concurrent chemotherapy. *FU* follow-up, *NAR* number of patients at risk, *CRT* chemoradiotherapy

**Fig. 2:**
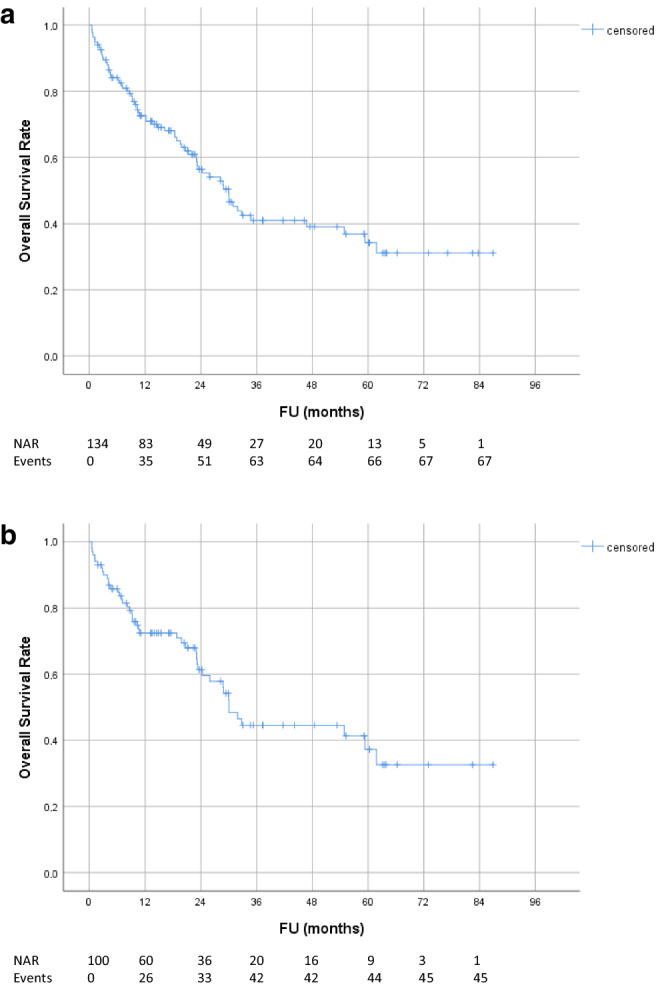
Overall survival: **a** all patients; **b** subgroup of patients with UICC stage III treated with concurrent chemotherapy. *FU* follow-up, *NAR* number of patients at risk

**Fig. 3:**
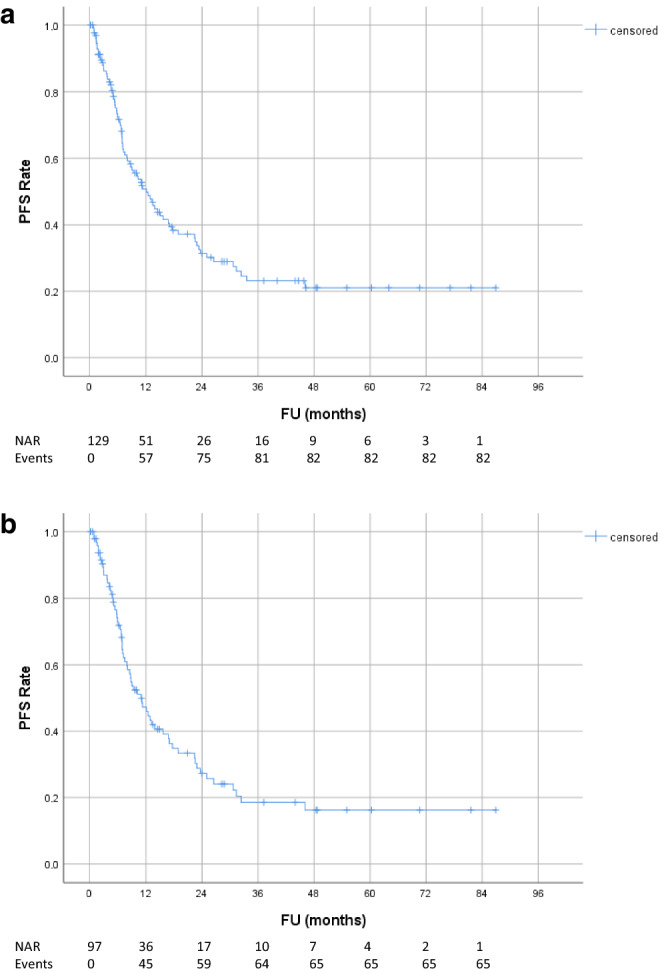
Progression-free survival: **a** all patients; **b** subgroup of patients with UICC stage III treated with concurrent chemotherapy. *PFS* progression-free survival, *FU* follow-up, *NAR* number of patients at risk

Median follow-up and radiological follow-up for all patients was 18.0 months (range 0.6–86.9) and 16.0 months (range 0.2–86.9), respectively. Local and regional tumor control rates are shown in Fig. 1. Local control rates at 1, 2 and 3 years were 80.4, 68.4 and 57.8%, respectively. Taking into account regional failures outside the RT field, the locoregional control rates at 1, 2 and 3 years were 66.6, 51.8 and 44.1%, respectively. Overall and progression-free survival rates are shown in Figs. 2 and 3: OS and PFS at 1, 2 and 3 years were 72.7, 56.4, 41.0% and 50.7, 31.4, 23.1%, respectively. Median overall and progression-free survival was 30.0 months (95% CI 23.5–36.4) and 12.1 months (95% CI 8.2–16.0), respectively. At the 5‑year FU, 71% of patients developed distant metastases. There was no statistically significant difference in OS (*p* = 0.30) or PFS (*p* = 0.76) between patients with or without oligometastasis. Moreover, local control between patients treated with CRT and patients treated with RT only did not differ significantly (*p* = 0.68). For the subgroup of UICC stage III patients that had been treated with concurrent CRT, local control rates at 1, 2 and 3 years were 77.9, 65.6 and 56.9%, respectively. OS and PFS at 1, 2 and 3 years were 72.5, 61.4, 44.6% and 47.2, 27.3, 18.5%, respectively (Figs. 1, 2 and 3). Median overall and progression-free survival in this subgroup was 30.0 months (95% CI 25.8–34.2) and 11.0 months (95% CI 7.5–14.5), respectively.

UICC stage (8th edition), a PTV SIB > 291 cm^3^, PTV and mean lung dose were statistically significant predictors of death on univariate analysis (Table 2). A PTV ≥ 600 cm^3^ was predictive for death on multivariate analysis. Univariate analysis revealed that UICC >III B, GTV > 133 cm^3^, PTV SIB > 221 cm^3^ and Karnofsky performance status (KPS) were predictive for a local recurrence. PTV SIB > 221 cm^3^ and KPS were statistical significant predictors of LR on multivariate analysis (Table 3).

**Table 2:** Risk of death

	Univariate				Multivariate			
Variable	*p* value	Odds ratio	95% CI		*p* value	Odds ratio	95% CI	
Age	0.13	–	–	–	–	–	–	–
Sex	0.58	–	–	–	–	–	–	–
UICC Stage 8th edition	**0.02**	1.45	1.06	1.97	0.15	–	–	–
UICC >III B	**0.01**	2.54	1.26	5.15	0.07	–	–	–
UICC IV	0.10	–	–	–	0.23	–	–	–
Total dose	0.16	–	–	–	–	–	–	–
Total dose > 68.7 Gy	0.47	–	–	–	–	–	–	–
GTV Volume	0.84	–	–	–	–	–	–	–
GTV > 212 cm^3^	0.11	–	–	–	–	–	–	–
PTV SIB volume	0.21	–	–	–	–	–	–	–
PTV SIB > 291 cm^3^	**0.03**	2.49	1.09	5.67	0.55	–	–	–
PTV	**<0.01**	1.00	1.00	1.00	0.71	–	–	–
PTV ≥ 600 cm^3^	**<0.01**	5.57	2.65	11.70	**<0.01**	5.18	2.40	11.21
Lungs D_mean_ (Gy)	**0.02**	1.10	1.01	1.20	0.46	–	–	–
Lungs D_mean_ > 15.44 Gy	**<0.01**	2.93	1.42	6.08	0.75	–	–	–
Simultaneous chemotherapy	0.10	–	–	–	0.46	–	–	–
KPS	0.10	–	–	–	0.27	–	–	–

*CI* Confidence interval, *UICC* Union for International Cancer Control, *Gy* Gray, *GTV* Gross tumor volume, *PTV* Planning target volume, *SIB* Simultaneous integrated boost, *KPS* Karnofsky performance status

**Table 3:** Risk of local recurrence

	Univariate				Multivariate			
Variable	*p* value	Odds ratio	95% CI		*p* value	Odds ratio	95% CI	
Age	0.07	–	–	–	0.11	–	–	–
Sex	0.75	–	–	–	–	–	–	–
UICC Stage 8th edition	0.07	–	–	–	0.09	–	–	–
UICC >III B	**0.04**	2.31	1.05	5.11	0.09	–	–	–
UICC IV	0.77	–	–	–	–	–	–	–
Total dose	0.07	–	–	–	0.44	–	–	–
GTV volume	0.68	–	–	–	–	–	–	–
GTV > 133 cm^3^	**<0.01**	3.07	1.34	7.04	0.35	–	–	–
PTV SIB	0.11	–	–	–	–	–	–	–
PTV SIB > 221 cm^3^	**<0.01**	3.83	1.69	8.71	**<0.01**	0.94	0.90	0.99
PTV volume	0.53	–	–	–	–	–	–	–
PTV > 294 cm^3^	0.08	–	–	–	0.83	–	–	–
Lungs D_mean_ (Gy)	0.07	–	–	–	0.69	–	–	–
Lungs D_mean_ > 16.7 Gy	0.22	–	–	–	–	–	–	–
Simultaneous chemotherapy	0.72	–	–	–	–	–	–	–
KPS	**0.03**	0.95	0.91	0.995	**0.03**	0.94	0.90	0.99

*CI* Confidence interval, *UICC* Union for International Cancer Control, *GTV* Gross tumor volume, *PTV* Planning target volume, *SIB* Simultaneous integrated boost, *Gy* Gray, *KPS* Karnofsky performance status

Radiation-induced pneumonitis grade 2 and grade 3 occurred in 13 (9.8%) and 3 (2.3%) patients, respectively. CTCAE ≥grade 3 anemia, leukopenia and thrombocytopenia was observed in 4.5, 23 and 2.8%, respectively. Two patients died of tumor bleeding during treatment, and 1 patient died of progressive lymphangiosis carcinomatosa on treatment. A few weeks after completion of chemoradiotherapy, 2 patients died of a fulminant bacterial pneumonia and 1 patient after suffering from acute renal failure following a protracted infection. One patient needed pneumonectomy after CRT because of a superinfected tumor cavern. Adverse events are shown in Table 4.

**Table 4:** Adverse events

	Grade 1	Grade 2	Grade 3	Grade 4	Grade 5
*Acute toxicity*					
Thrombocytopenia	32 (29.4%)	6 (5.5%)	3 (2.8%)	0 (0%)	0 (0%)
Leucopenia	13 (11.5%)	30 (26.5%)	20 (17.7%)	6 (5.3%)	0 (0%)
Anemia	56 (50.9%)	25 (22.7%)	5 (4.5%)	0 (0%)	0 (0%)
Renal insufficiency	18 (17.0%)	3 (2.8%)	0 (0%)	0 (0%)	1 (0.9%)
Dysphagia	56 (40.6%)	42 (30.4%)	15 (10.9%)	1 (0.7%)	0 (0%)
Radiation dermatitis	49 (35.5%)	19 (13.8%)	6 (4.3%)	0 (0%)	0 (0%)
Dyspnea	51 (37%)	12 (8.7%)	2 (1.4%)	0 (0%)	0 (0%)
Cough	71 (51.4%)	1 (0.7%)	0 (0%)	0 (0%)	0 (0%)
Fatigue	83 (60.1%)	10 (7.2%)	0 (0%)	0 (0%)	0 (0%)
Weight loss	66 (47.8%)	4 (2.9%)	0 (0%)	0 (0%)	0 (0%)
Nausea	25 (18.1%)	29 (21.0%)	1 (0.7%)	–	–
Emesis	13 (9.4%)	9 (6.5%)	0 (0%)	0 (0%)	0 (0%)
Radiation pneumonitis	6 (4.5%)	13 (9.8%)	3 (2.3%)	0 (0%)	0 (0%)
*Late toxicity*					
Dysphagia	14 (10.8%)	7 (5.4%)	0 (0%)	0 (0%)	0 (0%)
Skin/Soft tissue changes	9 (6.9%)	0 (0%)	0 (0%)	0 (0%)	0 (0%)
Dyspnea	36 (27.7%)	9 (6.9%)	0 (0%)	0 (0%)	0 (0%)
Cough	49 (37.7%)	0 (0%)	0 (0%)	0 (0%)	0 (0%)

## Discussion

IMRT with simultaneous integrated boost was introduced into clinical practice about two decades ago. The principle of irradiating the GTV and regions at high risk of microscopic tumor spread with an increased fraction size and regions at lower risk of tumor manifestation “electively” with a lower dose permits dose escalation and higher biological efficacy of hypofractionated therapy, while sparing the surrounding tissue. This technique was first used for head and neck tumors. In lung cancer, implementation of SIB techniques is considerably more difficult due to breathing motion, changing anatomic conditions resulting from increasing or decreasing effusion, atelectasis, changes in tumor size, large differences in density of lung and solid organs or tumor. To our knowledge, the presented cohort currently documents the largest number of patients treated for locally advanced NSCLC using this technique of SIB-IMRT. It shows that the SIB concept is applicable in practice with good success.

In current radio-oncological practice most commonly dose sparing to organs at risk is attempted by limiting the PTV to the primary tumor and the affected lymph node regions, an approach that became known as involved-field radiation therapy (IFRT) [10]. We recognize that there is growing evidence of IFRT not being inferior to elective nodal irradiation (ENI) with respect to locoregional failure [10–12]. However, IFRT regularly requires the addition of quite large margins to the GTV: the ESTRO guideline recommends the inclusion of the entire affected lymph node region, or at least the nodal GTV plus a 5–8 mm margin. To this CTV another 5–10 mm shall be added to generate the PTV. This volume should then be treated homogeneously with a high dose up to at least 60 Gy [13]. The limits of this concept are reached when large parts of the mediastinum or supraclavicular region would have to be irradiated with a high dose in multilevel N2 or N3 stages with multiple lymph node metastases. Compared to a homogeneous dose prescription, the presented SIB technique considerably lowers the volume of PTV that has to receive a dose of > 60 Gy in this group of patients, who accounted for a high proportion of our series: 46% of our patients had nodal stage N3. In our cohort ENI was still used. We are aware that many have abandoned this approach in favor of IFRT. However, due to the margins employed in published trials using IFRT, doses to organs at risk and side effects reported are not much different to our data [10–12]. Although the accuracy of FDG-PET with respect to TNM stage is quite high, the sensitivity to detect lymph node metastases on a per station basis is less than 60%, when compared to mediastinoscopic sampling [14]. Furthermore—as in all diagnostic tests—the negative predictive value of FDG-PET decreases with the prevalence of metastases. Therefore, there is probably an increased likelihood of false-negative findings when the PET scan already detects multiple lymph node metastases. Accordingly, the ESTRO guideline also recommends a rather “generous” inclusion of borderline positive findings in PET, which somewhat blurs the border between IFR and ENI. The difference in PTV between IFRT and ENI decreases in the presence of high stage nodal disease in the ipsi- and contralateral mediastinum. In this situation the combination of a simultaneous boost to the GTV with integrated sparing of organs at risk, both provided by SIB-IMRT may offer a relevant advantage, making a potentially curative treatment concept possible in cases where otherwise palliative radiotherapy would have been the only option. In line with this, Wang et al. found comparable outcomes for patients with locally advanced NSCLC treated by SIB-IMRT and for patients treated by conventional IMRT without SIB, although more patients with SIB-IMRT suffered from advanced N3 nodal disease [15].

SIB-IMRT with simultaneous chemotherapy led to excellent local tumor control in our patients. Though considering the observed regional failures outside the RT field and distant failures this good local control could not be transferred into convincing PFS rates, which corresponds to previous reports on CRT for locally advanced NSCLC [16]. Here, the combination of SIB-IMRT with consolidation immunotherapy with durvalumab as the present standard of care for patients showing no tumor progression contemporary after CRT hopefully will improve the outcome [3].

Obviously adoption of the new UICC classification effects relevant stage migration. Comparison to results from the literature may prove more difficult. In spite of a high proportion of UICC stage IIIC or IVA, a median survival of 30.0 months with a 5-year overall survival of 34% was achieved using a median total dose to the primary tumor of 63.8 Gy in 2.2 Gy fractions. This is a 2 Gy equivalent dose (EQD2) of 64.9 Gy for an α/β ratio of 10 Gy. It compares well with our preceding results using three-dimensional conformal chemoradiotherapy with a sequential boost to 66 Gy and no image guidance [2]. It is also in line with the findings of the RTOG 0617 trial that set the benchmark for chemoradiotherapy in NSCLC with a total dose of 60 Gy in 2 Gy fractions and found higher doses of 74 Gy being associated with worse local tumor control and survival [5, 17]. The authors stated that this unexpected result was probably caused by treatment-related deaths, treatment delays and concurrent chemotherapy abortions that were more common in the 74 Gy than in the 60 Gy arm. Also protocol deviations concerning treatment planning and target volume coverage by the 95% isodose were more frequent in the high-dose group. Heart dose was shown to be significantly related to survival in that study and led to the implementation of heart dose–volume constraints in consecutive trials. Also in the meta-analysis of Ramroth et al. including 3795 patients with NSCLC from 21 randomized trials higher radiation therapy doses led to poorer survival when concurrent chemotherapy was given, but in trials without chemotherapy, higher, time-corrected biologically equivalent doses resulted in longer survival [18]. Intensely escalated dose regimens can be safely applied while not combined with full-dose chemotherapy. Belderbos et al. showed good tolerability and median overall survival rates after RT with 66 Gy in 2.75 Gy fractions combined with low-dose cisplatin [19]. In the context of full-dose concomitant chemotherapy these findings support local dose escalation only in the combination with toxicity sparing. Type and implementation of radiotherapy could therefore play an important part. Jeter et al. reported on the feasibility and outcome of IMRT with SIB for patients with stage II–IIIB NSCLC receiving standard concurrent chemotherapy. In this prospective phase I dose-escalation study the authors found a SIB dose of 72 Gy in 30 fractions to be the maximum tolerated dose [20]. However, their reported case numbers are very low and FU time rather short, so one should await the results of their ongoing phase II trial. Li et al. reported on 20 patients treated with IMRT for inoperable stage II–III NSCLC with metastatic lymph nodes [21]. They treated their patients with a total dose of 78 Gy and 60–65 Gy for the primary and the metastatic lymph nodes, respectively, prescribed simultaneously in 26 fractions. Despite toxicity being commonly observed, the authors reported no grade ≥ 3 acute toxicity. By contrast, fiercely escalated hypofractionation to the primary tumor or subregions of high FDG-uptake in concurrent or sequential CRT has been shown to be feasible, but associated with higher acute and late toxicities compared to conventional CRT [22]. In our study, addition of full-dose concurrent chemotherapy did not result in excessive toxicity as only moderate dose-escalation/hypofractionation in combination with SIB-IMRT and IGRT was used.

Ma et al. conducted a retrospective study on SIB-IMRT in locally advanced NSCLC [23]. In a subgroup of their patients they had combined the increase of daily applied dose to the GTV by SIB-IMRT with an additional esophagus-sparing technique resulting in a reduction of radiation esophagitis without impairing locoregional control or overall survival. Likewise, in a secondary analysis of the NRG Oncology clinical trial RTOG 0617 IMRT was associated with lower rates of severe pneumonitis and cardiac doses [24].

Several factors may have contributed to the rather favorable outcome in our cohort. Nearly all patients had PET staging before radiation therapy. PET staging reduces the number of patients with undetected multiple distant metastasized disease that have no clear benefit from treatment with chemoradiotherapy [25, 26]. PET was also used for SIB-target volume delineation complementing 4D and 3D CTs. Supported by the accuracy of CBCT-based IGRT, metabolic active tumor lesions were reliably covered by the high-dose volume. Patients had replanning-CTs during the SIB-IMRT course and target volumes were adapted in case of relevant changes of the tumor or OAR anatomy.

In addition, SIB enabled the use of mild hypofractionation within the GTV and accelerated treatment. This might positively affect tumor control with regard to prevented tumor cell repopulation [16].

SIB reduces doses outside the target volume compared to sequential boosting. Accordingly, relatively low mean lung doses despite large PTVs were achieved in our patients. This transferred in a low rate of 12% for clinically relevant pneumonitis (mainly grade 2).

Logistic regression analysis revealed that a higher UICC stage, a higher PTV SIB, a higher PTV and mean lung dose were significantly associated with a higher risk of death on univariate analysis, and a higher PTV remained significant on multivariate analysis. We interpret these data as showing patients with locoregionally highly advanced tumor disease and multilevel nodal involvement at higher risk of death: in these patients with higher UICC stage, the multilevel nodal disease leads to higher volumes for PTV SIB and PTV and consecutively to higher mean lung doses. So the worse prognosis in these patients might probably be caused by the advanced tumor disease itself; however, causal influences by RT treatment parameters cannot be ruled out. Patients with UICC stage >III B, higher GTV, PTV SIB and lower KPS were at higher risk for local tumor recurrence on univariate analysis, and PTV SIB and KPS remained significant on multivariate analysis. Here again, advanced tumor disease associated with higher treatment volumes might itself inhere a higher risk of recurrence. A lower KPS being predictive for LR could be explained by the fact that these patients could not be treated as intensely as patients with better performance status by the addition of concurrent chemotherapy, as KPS was a major selection criteria for the application of concomitant chemotherapy. On the contrary, patients treated with RT alone showed no statistically significant difference with regard to local control in the log-rank comparison to patients treated with concomitant CRT. However, the subgroup of patients treated with RT alone was very small (10 patients) and the comparison of larger cohorts would probably favor CRT over RT. But we can conclude that SIB-IMRT without chemotherapy enables long-term local tumor control in patients not eligible for concomitant chemotherapy.

Some patients in our study had oligometastatic disease not exceeding two extrathoracic metastases. It has been shown that patients with limited metastatic disease benefit from (chemo)radiotherapy of the primary tumor in combination with metastasis-directed local treatment with regard to PFS and OS [27–31]. In our cohort we observed a median PFS of 7.0 months and a median OS of 19.6 months for the subgroup of UICC stage 4 patients. However, this subgroup is small and heterogeneous.

There are obvious limitations to our study. The retrospective design carries the risk of underestimating toxicities. Also consecutive systemic treatments may have affected survival. Patients were not treated to the present standard of consolidative durvalumab after CRT. Local failures frequently were detected using CT and not PET-CT. The substantial decline in patients at risk most commonly due to deaths but also due to losses to FU limits the validity of tumor control and overall survival assessment. Treatment-related impact on patients’ quality of life was not assessed. However, patients had regular follow-up and outcome/life status was updated at the time of data analysis for all patients.

Taking this into account: in this “real world” cohort of SIB-IMRT for the treatment of NSCLC from two departments’ clinical routine, treatment with SIB-IMRT was very effective and tolerable.

## Conclusions

Chemoradiotherapy by SIB-IMRT may result in excellent local tumor control. The overall survival appears promising regarding the high proportion of patients with UICC stage ≥IIIB disease. The SIB concept resulted in low mean lung doses and minor toxicity. In the wake of consolidative immunotherapy [3, 4], where lung tolerance is an issue, strict IMRT-IGRT protocols with tailoring high-dose areas to the GTV should be studied in prospective trials.
